# (Re-) conceptualising vulnerability as a part of risk in global health emergency response: updating the pressure and release model for global health emergencies

**DOI:** 10.1186/s12982-019-0084-3

**Published:** 2019-04-08

**Authors:** Charlotte Christiane Hammer, Julii Brainard, Alexandria Innes, Paul R. Hunter

**Affiliations:** 1 Norwich Medical School, University of East Anglia, Norwich, UK; 2 School of Philosophy, Politics and Language, University of East Anglia, Norwich, UK

**Keywords:** Vulnerability, Risk, Emergency response, Communicable diseases, PAR model

## Abstract

Vulnerability has become a key concept in emergency response research and is being critically discussed across several disciplines. While the concept has been adopted into global health, its conceptualisation and especially its role in the conceptualisation of risk and therefore in risk assessments is still lacking. This paper uses the risk concept pioneered in hazard research that assumes that risk is a function of the interaction between hazard and vulnerability rather than the neo-liberal conceptualisation of vulnerability and vulnerable groups and communities. By seeking to modify the original pressure and release model, the paper unpacks the representation or lack of representation of vulnerability in risk assessments in global health emergency response and discusses what benefits can be gained from making the underlying assumptions about vulnerability, which are present whether vulnerability is sufficiently conceptualised and consciously included or not, explicit. The paper argues that discussions about risk in global health emergencies should be better grounded in a theoretical understanding of the concept of vulnerability and that this theoretical understanding needs to inform risk assessments which implicitly used the concept of vulnerability. By using the hazard research approach to vulnerability, it offers an alternative narrative with new perspectives on the value and limits of vulnerability as a concept and a tool.

## Introduction

While health and medicine use the term “risk” widely, its use usually lacks conceptualisation and is often defined merely in the sense of probability. This approach may suffice for traditional individual and population health issues. However, in the context of health emergencies and disaster health, it could benefit from a more thoroughly conceptualized addition.

Global health emergency response operates along similar lines as global disaster and humanitarian response and often in concert with actors from these fields. Learning from the conceptual discussions underlying disaster studies and hazard geography perspectives does not only lend a new lens to understand risk differently but this more comprehensive approach would also facilitate risk management and risk reduction in global health emergency response and thus lead to a more sustainable response. This paper provides a possible pathway for answering the question how can disaster studies and hazard geography help us develop a (social) vulnerability theory for global health emergencies.

Therefore, this paper seeks to bridge the gap between the disaster studies literature and the medical understanding of risk and suggests the adaptation of a classic model for understanding risk from the disaster studies, the Pressure and Release (PAR) model [1] for global health emergencies. The PAR model is arguably the best known and most accepted model for conceptualizing risk in the context of disaster and emergency and offers a comprehensive and compelling framework for understanding the role of (social) vulnerability in risk. As such, this paper focuses mainly on the role of vulnerability, currently an under-conceptualized component of risk in health emergencies.

### Current uses of risk in health and medicine

Despite the mentioned lack of conceptualisation, risk is widely used in health and medicine and is a key element in epidemiology. Examples for the use of risk in health and medicine include risk ratios [2, 3], attributable risks [2, 3], diseases risks for individual patients and populations [2, 3], and comparisons of proportions of a population at risk [4]. In these contexts, “[r]isk has a very similar meaning in epidemiology as it does in everyday usage—it is about chance. It is defined by Unwin et al., as ‘the probability that an event will occur’. It is often used to compare the risk of an event between groups” [5]. While this non-conceptual definition has merit, especially in traditional highly quantitative approaches to population health, it also comes with limitations. It omits the role of vulnerability as a key component of risk and as such, impedes risk reduction in less quantitative and data-rich situations. This paper does in no way argue that all understandings of risk (or vulnerability) in health and medicine should be replaced by a new understanding, which is closer in line with that from disaster studies. Instead we argue that, in the case of global health emergency response, an additional understanding of risk could be helpful both to better identify risks and vulnerabilities and respond to them as well as to facilitate cooperation with other actors in order to achieve comprehensive mitigation and risk reduction strategies.

## Key concepts

While it goes beyond the scope of this article to give detailed definitions of all key concepts underlying both the original PAR model and the updated version, a short introduction to some of those concepts—namely hazard, vulnerability, risk and resilience—and their implication for the PAR model and its update is appropriate. The PAR model, in the tradition of disaster studies, rightly assumes risk to be more than just the possibility of an adverse event taking place and conceptualises risk as a function of hazard and vulnerability. This more complex conceptualisation also facilitates an understanding of resilience beyond that of a ‘bounce back (better)’ capacity.

### Hazard

Understanding hazard is at the same time the starting point for understanding risk and the least controversial part of risk in the context of the PAR model and of conceptualising risk. Hazard in this context is, in most cases, the natural component. Following the debates about the use and discontinuation of the use of ‘natural disaster’ [6–8], hazard can be understood as the only (potentially) natural component of disasters. Hazards exist in nature and society in all forms, including traditional natural hazards such as geo-hazards (e.g. earthquakes or volcanic eruptions), hydro-hazards (e.g. tsunamis or floods), or—in the context of this paper most important—biohazards (such as all disease-causing micro-organisms). A separate category in this context is technological hazards, which are not per se natural but driven by human action. The term and concept hazard does, however, make no comment about the level of risk these hazards pose to humans (or animals, the environment, society, or the economy for that matter). In order to understand the potential risk associated with a hazard the dimension of vulnerability is necessary.

### Vulnerability

Vulnerability lies at the heart of the conceptualisation of risk and of the traditional PAR model. Vulnerability is key component of risk and risk itself does not exist without vulnerability [9]. Vulnerability can be roughly defined as a function of exposure and susceptibility and can be applied to humans, environmental entities or societal or even technical structures.

$${\text{Vulnerability}} = {\text{Exposure}} \times {\text{Susceptibility}}$$

Most—if not all—elements traditionally in the medical, health and epidemiology field termed ‘risk factors’ fall within the category of vulnerability and can be either on the exposure or on the susceptibility side. “Susceptibility is a capacity characterizable by a set of intrinsic and extrinsic factors that modify the impacts of a specific exposure upon risks/severity of outcomes in an individual or population” [10] while exposure characterises the likelihood of an encounter with the disease-causing organism and the level or strength of this encounter.

Vulnerability in this context plays a part in both likelihood and severity of disease and disease outbreaks for both individual patients and entire populations. The introduction of the concept of vulnerability is not meant to replace the concept of a risk factor but rather to offer a better understanding of why risk factors are risk factors and the underlying mechanisms of these risk factors, as well as to offer approaches to reduce the risk of diseases by reducing (human) vulnerability.

### Risk

Risk is a complex concept made up of both hazard and vulnerability, even going beyond its components. Beck defines risk as “the modern approach to foresee and control the future consequences of human action, the various unintended consequences of radicalized modernization. It is an (institutionalized) attempt, a cognitive map, to colonize the future” [11]. While such a future oriented approach to risk is certainly beneficial in the context of resilience and of sustainable disaster and global health emergency response, the core of risk and the need for its conceptualisation in this context lies more within its ability to give different avenues to risk reduction by unpacking the interaction between hazard and vulnerability to form risk. As such, Ewald’s conclusion that “[n]othing is a risk in itself; there is no risk in reality” [9] still holds true and forms the very basis of vulnerability and hazard and their distinction from risk.

Considering the traditional conceptualisation of risk as a function of both hazard and vulnerability, which also forms the basis of the traditional PAR model [1] risk is often defined as the following:

$${\text{Risk}} = {\text{Hazard}} \times {\text{Vulnerability}}$$

Combining this equation with the above introduced equation for vulnerability leads to a complex understanding of risk:

$${\text{Risk}} = {\text{Hazard}} \times {\text{Exposure}} \times {\text{Susceptibility}}$$

This is not necessarily meant as a quantifiable equation but rather as a conceptual backdrop for understanding risk and its components. However, one fundamental mathematical truth plays a crucial role in this equation. The idea that without hazard or without vulnerability there is no risk is central to both the understanding of risk and the use of the traditional PAR model as well as any adaptation for global health emergency response. The hazard side of the equation is less of a focus for the PAR model and thus possibilities for hazard reduction are not prioritized. However, within the PAR model, a significant reduction in vulnerability leads to a significant reduction in risk and a (however hypothetical) eradication of vulnerability leads to an eradication of risk. Being able to reduce risk by being able to target multiple different aspects of it gives additional options for risk reduction, mitigation and risk management.

### Resilience

While definitions of resilience are highly contested [12] and the benefit and potential harm of the concept of resilience itself has been debated in the context of neoliberal society [13–15], all definitions of resilience carry with them at least some aspects of absorbing, changing and carrying on [16] as well as of recovery [17]. These ideas are often augmented by conceptualisations about resistance, absorption and restoration [6] and the ability to ‘bounce back’ [18] or even to emerge stronger. Schoon describes resilience as “a two-dimensional construct defined by the constellations of exposure to adversity and the manifestation of successful adaptation in the face of that risk” [19]. As such, a complete conceptual understanding of risk, including its components is, if not necessary, then at least highly beneficial to understanding and thus actively fostering resilience. Active disaster risk reduction enhances resilience. This holds true for global health emergencies as much as for other disasters. While reducing the hazard (the disease-causing organisms) is an admirable intention, it is also highly dependent on the specific type of bio-hazard. Focusing on the vulnerability side has the advantage of also offering perspectives for situations of unknown hazards. Thus, there is a need to increase focus on the vulnerability side of the risk—including both susceptibility and exposure to the hazard. This approach holds the greatest promise of producing enduring resilience and therefore to a sustainable global health emergency response.

## The original pressure and release (PAR) model

The original PAR model follows the understanding of risk as a function of hazard and vulnerability and focuses on the vulnerability side of risk and especially on factors related to susceptibility. While not clearly conceptualised, the original PAR model does include aspects of exposure but it does not directly associate these with susceptibility as a part of vulnerability. This could be seen as a critique of the original model. Due to the slight differences of global health emergencies to disasters associated with natural hazards, our adapted version explicitly includes aspects of heightened exposure in the progression of vulnerability.

### Components of the original PAR model

The original or traditional PAR model defines three steps to explain the progression of vulnerability: root causes, dynamic pressures and unsafe conditions [1]. Each step in the progression of vulnerability builds on the step(s) before and leads to increasing pressure on the whole system. These steps, combined with the presence of hazard, lead to risk of disaster and ultimately to disaster [1]. Root causes in the original PAR model include limited access to power, limited access to structures, limited access to resources, aspects of the political system(s) and aspects of the economic system(s) [1]. Root causes as such, are at the structural level and often describe underlying situations and power dynamics that are ingrained in a society or group. According to the original PAR model, these root causes can then lead to dynamic pressures, which include lack of training, lack of local investment, lack of press freedom, rapid population change, rapid urbanisation, and deforestation [1]. Root causes are mainly static and resistant to change within the span of an emergency response. Dynamic pressures are evolving systems that can lead to increasing pressure and subsequently to unsafe conditions. Unsafe conditions include the physical environment, the local economy, social relations and public actions [1]. They are, in terms of traditional health and medical terminology, the most immediate risk factors. However, their causes lie in the preceding steps of the progression of vulnerability [1].

### Critique of the original PAR model

As mentioned before, the role of exposure is not entirely clear in the original PAR model, however, it is sufficiently clear for the original uses. While the original model also lists ‘viruses and pests’ as potential hazards, the progression of vulnerability for those is slightly different. Most of the original factors and steps still hold true but they are insufficient to explain the progression of vulnerability towards disaster, which in this case can be defined as the outbreak of a disease, hence making an adaptation especially for global health emergencies sensible.

Other critiques of the original PAR model focus mainly on its lack of environmental focus, either expressed as a lack of focus on the role of sustainability [20] or as a lack of focus on human–environment interactions and the vulnerability of the biophysical world [21]. However, these issues have since been addressed in the second version of the model. We acknowledge that the original PAR model—and the adapted version presented in this paper as well—certainly still has a decidedly human focus, specifically a focus on human vulnerability with an underlying assumption that socio-economic vulnerability is key to risk. It is our aim to broaden the perspective on global health emergency response and a broader, adapted PAR model is one component of this.

## The updated PAR model for health emergencies

**Fig. 1:**
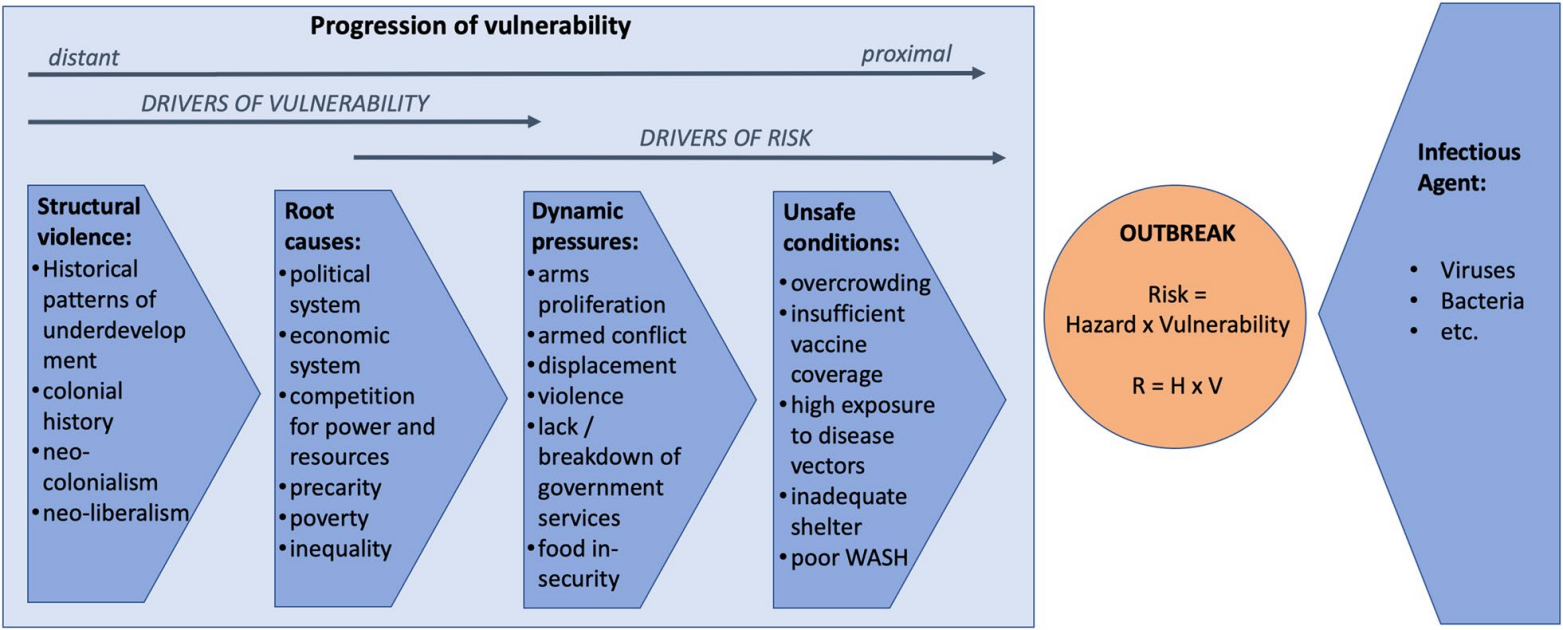
Adapted PAR model for health emergencies

While many of the assumptions made in the context of the original PAR model still hold true for a health specific update, they need to be critically examined and in some places augmented by root causes, dynamic pressures, and unsafe conditions that are more specific to health risk. The improved understanding of the progression of vulnerability in health emergencies has implications for vulnerability, risk and resilience and their conceptualisation—and lack thereof—in the concept of health emergencies (Fig. 1).

### Components

While the traditional root causes (limited access to power, structures and resources, and political and economic systems) certainly hold true in the context of health emergencies the related issues of competition for power and resources [1], precarity [22, 23], poverty [22, 24, 25], and inequality [22, 24, 25] warrant further emphasis as root causes that facilitate the development of dynamic pressures. Competition for power and resources could be interpreted as a part of limited access to power, structures and resources. However, the level at which those root causes act and interact is different. Limited access to power, structures and resources arises from lack of an inclusive and democratic society and political system. Competition for power and resources does not necessarily assume widespread access to power and resources. It focuses on those groups and individuals who have access and on how their interaction stabilises or destabilises any given situation. Additionally, we suggest considering environmental and ecological fragility. Environmental and ecological fragility describes the resilience or lack thereof of the natural environment and hence plays an important part in characterizing the geographical context. While it is not a component of social vulnerability, environmental fragility strongly impacts severity of exposure.

We see all of these root causes as based on conditions of structural violence comprised of historical patterns of underdevelopment, colonial histories, neo-colonialism, and neo-liberalism, which act as drivers of vulnerability and form an integral part of the early progression of vulnerability [22]. These forms of structural violence and their ingrained stigmatization and marginalization of populations along lines of race, ethnicity, religion, gender, sexuality, and socioeconomic status, lead to historically-rooted inequalities, which form the backdrop of many of the root causes, dynamic pressures, and unsafe conditions.

For dynamic pressure, the updated PAR model for health emergencies does not negate the importance of the originally stipulated dynamic pressures (lack of training, lack of local investment, lack of press freedom, rapid population change, rapid urbanisation and deforestation). However, if the risk that is being examined is that of a health crisis more suitable dynamic pressures can be found and substantiated by the evidence. We suggest the following dynamic pressures: arms proliferation, armed conflict, displacement, violence, lack or breakdown of government services, lack of access to health care, and food insecurity. Arms proliferation is a direct precursor to armed conflict, which is arguably one of the main drivers for health emergencies that are secondary to a humanitarian crisis. Armed conflict and violence together foster a climate of insecurity which is conducive to disease outbreaks through a variety of mechanisms [26–35]. Population displacement leads to a lack of access to health services [27, 30, 36–38] and generally unsafe living conditions, both in camp and community settings [26, 27, 29–32, 34–47]. A lack or breakdown of government services can lead to a breakdown of health related infrastructure including individual health services and population health services such as vaccination [26, 27, 29, 30, 32–36, 38–45, 47–49] as well as a breakdown of other (critical) infrastructure and coordination [30, 32, 34, 35, 37, 50]. All of the preceding can produce health emergencies. Food insecurity can be seen as a key precursor to malnutrition which is an important risk factor, both at the level of population and at the individual level, for communicable diseases [27, 30, 32, 36, 38–42, 49–51] and other health conditions [52–54].

In terms of unsafe conditions, we propose inclusion of the following, which are all highly conducive to ill health and direct or indirect progressions of the aforementioned dynamic pressures: overcrowding, insufficient vaccine coverage, high exposure to disease vectors, inadequate shelter, and poor water, sanitation and hygiene (WASH). Overcrowding, which can result from both displacement and entrapment, facilitates the spread of diseases from person to person and is thus a key risk factor for communicable disease outbreaks [27, 30, 32, 35–39, 42, 49, 50, 55–67]. Insufficient vaccine coverage is produced both by a breakdown of government services, especially population health services, and by unvaccinated persons being displaced into areas with higher disease prevalence. Absence of vaccination has for example been identified as an unsafe condition in the example of the European migration crisis [61, 62, 64, 68–70]. Similarly, increases in the presence of disease vectors, such as specific species of mosquitos the likelihood of an outbreak and of the transmission of vector-borne diseases [29, 61] have significant consequences. Inadequate shelter without proper heating, ventilation and cooking facilities has implications both for communicable diseases [30, 32, 35, 39, 41, 56, 60–62, 66, 71] and for non-communicable health such as asthma and COPD especially if indoor fires are used [30, 32, 39]. Finally, the role of poor WASH as a risk factor and as such, as an adequate unsafe condition for communicable diseases, has been well documented [27, 29, 30, 32, 35–39, 41, 49–51, 59, 60, 63, 66, 67, 72–75].

What we traditionally call a risk factor in health, medicine and epidemiology is - according to the model and seen in a more complex picture—in fact a stage in the progression of vulnerability or in other words a component of the overall vulnerability. Vulnerabilities are what might lead to disease in an individual and to an outbreak or epidemic in a population.

### Implications for the understanding of vulnerability and risk in health emergencies

This model follows the original PAR model [1] in its understanding of (the progression of) vulnerability. As such, vulnerability becomes a function of root causes, dynamic pressures and unsafe conditions:

$${\text{Vulnerability}} = {\text{Root Causes }} \times {\text{ Dynamic Pressures }} \times {\text{ Unsafe Conditions}}.$$

Vulnerability and its progression stem from these multiplicatory components. The model highlights the interaction and progressive nature of the system. Those components traditionally identified as risk factors for health emergencies are most commonly found in the third category, unsafe conditions. While these are undoubtable the most direct risk factors, focusing only on them risks overlooking the complex causes of these unsafe conditions or risk factors.

The risk from the original equation in this context is the health emergency. That means, in many cases, an outbreak of a communicable disease, either as a stand-alone event such as the 2014 West Africa Ebola outbreak, or a larger humanitarian crisis, such as in ongoing Cholera epidemic in Yemen. When considering the original equation of risk being a function of hazard and vulnerability, the model and its components as described cover the vulnerability side, with the hazard being the disease-causing micro-organism. Recall Ewald’s conclusion that risk only exists with vulnerability [9]. This means that, while it is improbable that all vulnerabilities in situations such as the ones mentioned above can be reduced to zero, the risk can be greatly reduced by reducing the vulnerability towards said risk. This can be done without always needing a ‘toolkit’ to reduce hazard. Hazard reduction is a suitable method in some circumstances but it is not the only or necessarily most productive approach in all situations.

### Implications for the understanding of resilience in health emergencies

Understanding risk in terms of hazard and vulnerability fosters increased understanding of how to introduce and increase resilience by sustainably reducing vulnerability and therefore risk. Complex understandings of risk are a first step to work towards resilience, therefore our model may offer benefits. Our new concept of risk and vulnerability may highlight pathways to the ability to absorb, change, carry on [16], recover [17], resist, or absorb [12]. It is worthwhile to explore if the reconceptualisation can help lead to an increased capacity to ‘bounce back’ [18] or even bounce back better. However, more than just conceptual insights are needed in order to foster lasting and positive resilience. In the context of global health emergencies, the insights into vulnerability certainly highlight and reinforce that a focus on strengthening health systems can lead to a reduction of vulnerability and therefore a reduction of risk. Additionally, we believe that the dynamic element of the PAR model allows for the consideration of changing conditions—and the causes of the changes, as traceable through the progression of vulnerability—to be considered in both epidemiology and risk assessment, which allows for both mitigation and preparedness.

### Possible uses and advantages of the updated PAR model for health emergencies

Updating the original PAR model for health emergencies and using it in this context could lead to an improvement of the conceptual and practical understanding for the progression from population-level risk to outbreaks and epidemics. It could become easier to understand how a situation progresses to become an emergency. This prospect has direct and indirect implications for risk assessments, leading to potentially longer lead times between the detection of an increased risk due to increased vulnerability and an actual outbreak or epidemic.

Additionally, such a conceptual understanding can be used as a basis for improving targeted risk management and risk reduction interventions by providing action points for intervention and understanding where they lie in the progression of vulnerability. This opens the possibility to prioritise interventions.

Combining these two approaches leads to a potential use of the adapted PAR model for estimating risk and vulnerability under alternative management approaches. These could include scenario planning or forecasting as well as post hoc analysis in order to better understand the value and reasoning for decisions made. This is particularly relevant in contexts where situations are changing rapidly and creating considerable uncertainty. Thus, the adapted PAR model offers insights to facilitate *adaptive management:* adaptive strategies that develop in response to uncertain and changing circumstances.

Finally, harmonising the language of health emergency response with the language of disaster response can help foster a common understanding of concepts and facilitate better communication across sectors and clusters.

### Limitations

Different thinking and practical implications of reconceptualising vulnerability and risk in the context of health emergencies are difficult because risk is an ingrained concept in health and medicine. Moreover, the model does not offer automatic solutions or risk reduction measures. Instead, it seeks to contribute to a discussion on terminology and the implications of terminology for understanding, analysis, and action.

As it is currently built, the updated PAR model might be most suited to situations where general context and vulnerability progression are the focus rather than development of the hazard. Hence, the model might be more immediately and obviously suitable to explain the development of risk in cases of secondary health emergencies rather than emerging disease threats. It might be more suitable as an explanatory model for disease outbreaks in existing humanitarian crises such as the Cholera outbreak in Yemen rather than situations in which the disease outbreak constitutes the humanitarian crisis, such as the 2014 West Africa Ebola epidemic. We hope that use of our model will improve understanding of outcomes and add perspectives that acknowledge that underlying social complexity. The progression of vulnerability remains a pivotal aspect in both types of events. With regard to emerging disease threats, the model would explain only part of the problem and need to be augmented by understanding other concurrent processes regarding the evolution and progression of the hazard.

## Conclusion

Vulnerability is a key part in risk and this should be recognised in all fields that inherently deal with risk. While traditional definitions and terms such as ‘risk factor’ do not need to be replaced in the context of health and medicine, in global health emergency response, a more thorough consideration of their components certainly helps to understand mechanisms and pathways of risk beyond probability. This paper offers a theoretical model for renewed thinking about the meaning of risk and resilience and at the same time seeks to reconcile the language of health and medicine with the language of disaster studies and disaster response. The analysis of risk factors, augmented with the conceptual understanding of their place in the progression of vulnerability, is an important part of understanding how global health emergencies evolve. The theoretical backing tentatively offered in this paper supports quantitative study of the epidemiological basis for risk factors in individual emergencies by providing a wider understanding of the role of risk factors. We also argue strongly for an interdisciplinary approach to global health emergency response. This approach can open new avenues for mutual understanding.

## Abbreviation

PAR: pressure and release
